# Leukotriene A_4_ Hydrolase Is a Candidate Predictive Biomarker for Successful Allergen Immunotherapy

**DOI:** 10.3389/fimmu.2020.559746

**Published:** 2020-11-24

**Authors:** Ting-Ting Ma, Meng-Da Cao, Rui-Li Yu, Hai-Yun Shi, Wei-Jun Yan, Jian-Guo Liu, Chen Pan, Jinlyu Sun, Qing-Yu Wei, De-Yun Wang, Ji-Fu Wei, Xue-Yan Wang, Jin-Shu Yin

**Affiliations:** ^1^ Department of Allergy, Beijing Shijitan Hospital, Capital Medical University, Beijing, China; ^2^ Research Division of Clinical Pharmacology, The First Affiliated Hospital of Nanjing Medical University, Nanjing, China; ^3^ Department of Allergy, Duolun People’s Hospital, Duolun, China; ^4^ School of Basic Medicine and Clinical Pharmacy, China Pharmaceutical University, Nanjing, China; ^5^ Department of Allergy, Peking Union Medical College Hospital, Peking Union Medical College, Chinese Academy of Medical Sciences, Beijing Key Laboratory of Precision Medicine for Diagnosis and Treatment on Allergic Diseases, Beijing, China; ^6^ Department of Allergy, General Hospital of Northern Theater Command, Shenyang, China; ^7^ Department of Otolaryngology, Yong Loo Lin School of Medicine, National University of Singapore, Singapore, Singapore

**Keywords:** allergic rhinitis, allergen immunotherapy, serum, biomarkers, proteomics, LTA_4_H

## Abstract

**Background:**

Allergic rhinitis is a common disorder that affects 10% to 40% of the population worldwide. Allergen immunotherapy (AIT) represents the only therapy that has the potential to resolve clinical symptoms of allergic rhinitis. However, up to 30% of patients do not respond to AIT. Biomarkers predicting the clinical efficacy of AIT as early as possible would significantly improve the patient selection and reduce unnecessary societal costs.

**Methods:**

*Artemisia* pollen allergic patients who received at least 1-year AIT were enrolled. Clinical responses before and after 1-year AIT were evaluated to determine AIT responders. *Artemisia* specific IgE and IgG4 levels were measured by using ImmunoCAP and enzyme-linked immunosorbent assay (ELISA) separately. Stepwise regression analysis was performed to identify which rhinitis-relevant parameters explained the most variability in AIT results. Liquid chromatography-tandem mass spectrometry (LC-MS/MS)-based proteomics was applied to identify the potential candidate biomarkers in the sera of responders and non-responders collected before and after 1-year therapy. The diagnostic performance of the potential biomarkers was then assessed using enzyme-linked immunosorbent assay (ELISA) in 30 responders and 15 non-responders.

**Results:**

*Artemisia* specific IgE and IgG4 levels were elevated only in the responders. Regression analysis of allergic rhinitis-relevant parameters provided a robust model that included two most significant variables (sneeze and nasal congestion). Thirteen candidate biomarkers were identified for predicting AIT outcomes. Based on their association with allergy and protein fold change (more than 1.1 or less than 0.9), four proteins were identified to be potential biomarkers for predicting effective AIT. However, further ELISA revealed that only leukotriene A_4_ hydrolase (LTA_4_H) was consistent with the proteomics data. The LTA_4_H level in responders increased significantly (P < 0.001) after 1-year therapy, while that of non-responders remained unchanged. Assessment of LTA_4_H generated area under curve (AUC) value of 0.844 (95% confidence interval: 0.727 to 0.962; P < 0.05) in distinguishing responders from the non-responders, suggesting that serum LTA_4_H might be a potential biomarker for predicting the efficiency of AIT.

**Conclusion:**

Serum LTA_4_H may be a potential biomarker for early prediction of an effective AIT.

## Introduction

Allergic rhinitis is a common disorder that affects 10% to 40% of the population worldwide (1). It is defined as symptoms of sneezing, rhinorrhea, nasal pruritus and airflow obstruction (2). Allergic rhinitis may occur at any age, peaking in the teenage years (3). It also underlies many complications and is a major risk factor for poor asthma control (4–6). Uncontrolled allergic rhinitis has a negative impact on social life, work productivity and school performance, particularly in patients with severe symptoms. However, allergic rhinitis is often ignored, misdiagnosed, and mistreated, which can be detrimental to health and increases societal costs (3, 7).

Pollens are the major cause of seasonal allergic rhinitis. In northern China, over 50% patients with respiratory allergies are sensitized to *Artemisia* pollen (8). Many classes of drug are available to relieve allergy symptoms, including intranasal corticosteroids and antihistamines. However, even with the best pharmacotherapy, 20% affected individuals remain highly symptomatic (3). Allergen immunotherapy (AIT) represents the only available cause-oriented therapy so far that has the potential to alter the natural history of allergic rhinitis (9–12). AIT is defined as the repeated administration of specific allergens to patients with IgE-mediated allergies in order to provide protection against allergies and inflammatory reactions associated with natural exposure to the same allergens. It is often accompanied by an initial early increase in allergen-specific IgE (sIgE) and allergen-specific (sIgG4) levels (13). The exact mechanism by which AIT conveys a clinical benefit is unknown. Although efficacy of AIT has been demonstrated against highly prevalent allergens such as pollens and house dust mites, up to 30% of patients do not respond to it (14). No predictive biomarkers or diagnostic tests have been developed for its efficacy (15). The unpredictable outcome and a long course (up to 5 years) (16) of AIT imposes a high or even unnecessary cost to the health system especially for people who turn out to be non-responder. Therefore, early prediction of AIT efficacy would greatly reduce the overall cost associated with the treatment.

Clinical analysis of serum is the most widespread diagnostic procedure in medicine, and serum biomarkers are usually used to categorize patients and to support treatment decisions (17). Due to its availability and stability, serum represents valuable resource for proteome analysis and biomarker discovery (18, 19). This biological specimen is considered a key source of physiological information about the overall status of each tissue, and it reflects the status of several pathological conditions. The assessment of the circulating molecules, e.g., proteins, in serum may present a potential approach to help predict the outcome of AIT (12, 20).

**Figure 1:**
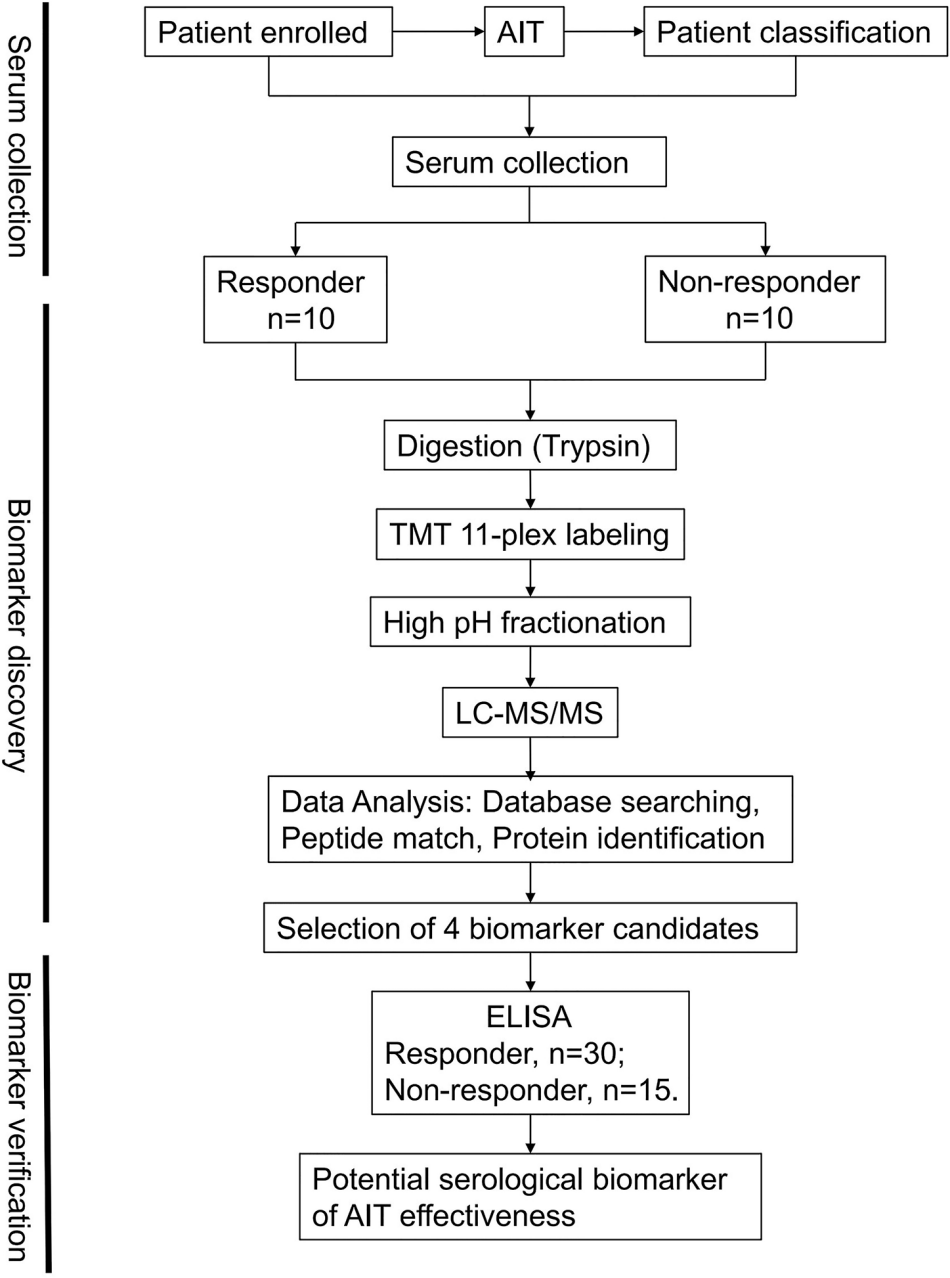
Schematic workflow. An overview of the workflow used for the identification of allergen immunotherapy (AIT) efficacy biomarkers.

Liquid chromatography-tandem mass spectrometry (LC-MS/MS)-based proteomic analysis can measure global protein abundance and post-translational modifications to provide additional biological insights (21) and rapidly identify candidate biomarkers in clinical samples (22). In this study, we used LC-MS/MS to profile the blood samples from patients after AIT to identify potential biomarkers and further validated the results using enzyme-linked immunosorbent assay (ELISA) (**Figure 1**). The outcomes of this work might help develop serum-based early assay for AIT efficacy using validated biomarkers and may help reduce the overall therapeutic cost and improve the outcomes.

## Methods

### Patients and Samples

Patients from 6 to 60 years of age with *Artemisia* pollen allergic rhinitis were recruited at Beijing Shijitan Hospital, affiliated to Capital Medical University on 28-31 July 2016 with a course of disease more than 12 months. Clinical responses were evaluated before and after AIT, including total nasal symptoms scores (TNSS), rhinoconjunctivitis quality of life questionnaires (RQLQ), VAS and allergic conjunctivitis symptoms. According to the treatment results after one year, patients were classified as responders (n = 30) and non-responders (n = 15). The curative effect was evaluated by symptoms and sign scores. The curative effect index (%) = (pretreatment total score − posttreatment total score)/pre-treatment total score × 100%. Patients with ineffective therapeutic index (≤25%, n = 15) were included the ineffective group. And those with effective therapeutic index (≥66%, n = 30) were classified as the effective group. Patients with not significantly effective or ineffective results (>25%, <66%, n=3) were not included in the analysis because of unclear treatment results.

Ten ml fresh blood was collected from the patients before AIT, immediately centrifuged at 1500 rpm at 4°C for 10 min and stored at −80°C for use in subsequent LC-MS/MS and ELISA. Then, serum was collected and processed in the same way before the last injection of 1-year AIT. Serum samples from 20 patients (10 AIT responders, and 10 AIT non-responders) with *Artemisia* pollen allergic rhinitis were used for comprehensive proteome profiling. For further development of potential biomarkers, ELISA verification was performed on an independent set of 45 pairs of serum samples (30 AIT responders, and 15 AIT non-responders). Characteristics of enrolled patients were summarized in **Table 1**. The study was conducted under the guidance of the Helsinki Declaration and approved by the Institutional Review Board of Beijing Shijitan Hospital, affiliated to Capital Medical University, Beijing, China. Written informed consent was obtained from every participant in this study.

**Table 1:** Baseline characteristics of patients.

	Effective (n = 30)	Ineffective (n = 15)	P
Age, years	27.4 ± 14.33	36.2 ± 16.26	0.07
Gender, n (%)			
Male	17(56.67)	13(86.67)	
Female	13(43.33)	2(13.33)	
Height, cm	161.27 ± 13.03	168.6 ± 13.65	0.09
Body weight, kg	56.63 ± 17.36	66.33 ± 22.64	0.12
Blood pressure, mm Hg			
Systolic	109.36 ± 15.11	118.67 ± 5.16	0.03
Diastolic	77.36 ± 10.66	84.33 ± 5.63	0.02
Heart rate, beats/min	77.25 ± 5.46	74.27 ± 3.53	0.06
Body temperature, °C	36.44 ± 0.16	36.43 ± 0.24	0.92
Breath rate, times/min	17.75 ± 1.94	17.2 ± 1.78	0.37
TNSS			
Sneeze	2.57 ± 0.57	2.27 ± 0.7	0.154
Rogue	2.63 ± 0.61	2.67 ± 0.49	0.941
Nasal congestion	2.37 ± 0.81	2.33 ± 0.9	0.989
Nasal itching	2.5 ± 0.78	2.07 ± 0.8	0.053
Total score	10.07 ± 1.53	9.27 ± 1.91	0.116
VAS score			
Patient evaluation	7.79 ± 1.31	7.24 ± 1.31	0.181
Doctor evaluation	7.74 ± 1.26	6.97 ± 1.2	0.05
Allergic conjunctivitis symptoms scores			
Itchy/redness	2.6 ± 0.62	2.4 ± 0.74	0.354
Tears	1.7 ± 1.02	1.67 ± 0.82	0.820
Total score	4.27 ± 1.34	4.13 ± 1.3	0.764
Intradermal test of Artemisia	3.97 ± 0.18	3.87 ± 0.52	0.590

### Inclusion Criteria

The inclusion criteria were as follows: (1) age between 6 and 60, (2) course of disease more than 1 year, (3) typical symptoms appearing in the summer and autumn pollen season, and asymptomatic or mild symptoms in the non-pollen season (non-pollen season VAS <3), (4) intradermal test of *Artemisia* pollen allergen was ≥+++, and sIgE was ≥II (based on UniCAP allergen-specific IgE detection system), (5) other types of allergen skin test was negative, or “+” and above (including “+”) but specific IgE was <II level. Other pollen allergen specific IgE (including pollens of ragweed, *Humulus*, *Chenopodium*, sunflower and oilseed rape) were at least two grades below the *Artemisia* pollen test. More specifically, if a wheal was 1/3 times as large as the histamine positive control, the result was defined as +; if a wheal was 2/3 times as large as the histamine positive control, the result was defined as ++; if a wheal was as large as the histamine positive control, the result was defined as +++; if a wheal was larger than the histamine positive control, the result was defined as ++++. The 0.7–3.5 kU/L sIgE was regarded as II level.

### Exclusion Criteria

The exclusion criteria were as follows: (1) simple rhinoconjunctivitis with TNSS<6, (2) forced expiratory volume in 1 s <75% during non-pollen season, (3) with nasal polyps, significant vasomotor rhinitis, severe asthma, malignancy, severe cardiovascular diseases and impaired renal function, etc., (4) having accepted AIT, (5) be using β-blockers, (6) cannot accept regular follow-up, (7) smokers of 30 packs-per-year or more, (8)be pregnant or plan to become pregnant in the next two years.

### Allergen Immunotherapy

AIT was initiated after confirmation of *Artemisia* sensitivity. At the build-up phase, patients were injected standardized *Artemisia* sieversiana pollen allergen extract with 1:10^12^ dilution (purchased from Beijing MacroUnion Pharmaceutical Limited Corporation, Beijing, China, batch number: S20130001, total protein content 1.75 mg/5 ml) for 10 times starting from 0.1 ml at the first time with an increment of 0.1 ml per time. The concentration of allergen extract was increased by 10 times each time, from 1:10^12^ dilution to 1:100 dilution of the original concentration, then reaching the maintenance dose. In the build-up phase, subjects received each concentration 10 times for 5 weeks from 0.1 to 1 ml with an increment of 0.1 ml per time. During the maintenance phase, the concentration was no longer increased, 0.5 ml was injected twice a week. Patients received AIT during the pollen season.

### Serum Immunoglobulin Analysis

Before AIT initiation and after 1-year therapy, *Artemisia*-sIgE levels were measured by using ImmunoCAP (Thermo Fisher Scientific, Uppsala, Sweden) according to the manufacturer’s protocols. The concentrations of *Artemisia*-sIgG4 in sera were also measured by a sandwich ELISA. Briefly, 96-well plates (467320, Nunc, Denmark) were coated overnight at 4°C with 100 μl of allergen extracts (XP61D3A2.5, Stallergenes Greer, USA) at a 1:1,000 dilution. Test wells were incubated with 10 μl of the sera sample and 40 μl 1% BSA/PBS (bovine serum albumin dissolved in phosphate-buffered saline at the concentration of 1%), while control wells were incubated with 50 μl 1% BSA/PBS. After adding secondary antibody (Mouse Anti-Human IgG4 pFc, 9190-05, Southern Biotech, USA)at a 1:4,000 dilution and 3,3′,5,5′-tetramethylbenzidine (TMB, PR1210, Solarbio, China), sIgG4 was determined at 450 nm using an ELISA plate reader (SPARK 10 M, Tecan, Switzerland). Antibody levels in the sera were quantified by extrapolation against the standard curve.

### Filter-Aided Sample Preparation and Digestion

For protein extraction, the sodium dodecyl sulfate (SDS) lysis buffer was added into the samples. Proteins were separated and digested by SDS polyacrylamide gel electrophoresis. However, SDS removal is critical before mass spectrometry analysis due to its ability to contaminate liquid chromatography systems and dominate mass spectra. Filter-aided sample preparation (FASP) is the most popular method for detergent removal and protein digestion in recent years (23). FASP was performed according to a previously described method with slight modifications (24). Briefly, 100 µg proteins were moved into a EP tube and centrifuged at 12,000×g for 30 min. One microliter M dithiothreitol (DTT) was added and the sample was incubated for 2-3 h at 37°C. The digested sample was loaded to a 30 k Microcon filtration device (Millipore, Darmstadt, Germany) and centrifuged for 40 min at 12,000×g. The concentrate was then mixed with 100 µl 50 mM iodoacetamide (IAA) and incubated for 30 min in darkness at room temperature. IAA was removed by centrifugation at 12,000×g for 40 min. The sample was washed with 100 µl triethyl ammonium bicarbonate (TEAB) for four times and digested with trypsin (Promega) at a trypsin: protein ratio (w/w) of 1:50 in 50 µl TEAB overnight and followed by another 4 h digestion at 37°C. The tryptic peptides were collected by centrifugation at 12,000×g and desalted using StageTipC18 purification system.

### Isobaric Tandem Mass Tags Labeling and High pH Fractionation

Peptides were reconstituted in 100 mM TEAB and concentration was determined by the bicinchoninic acid (BCA) assay. Total of 40 samples were divided into 4 groups. Each group contained 10 samples (5 pairs) and 1 internal reference. For the ‘‘internal reference’’ mixed sample used in tandem mass tags (TMT) labeling, all the samples in same group were mixed in equal protein amount, and labeled using TMT 10 isobaric label reagent and TMT11-131C label reagent (Thermo Fisher Scientific). Each sample containing 30 μg peptide in 50 μl TEAB buffer was combined with 41 μl of respective TMT-labeled reagent and this mixture incubated for 1 h at room temperature. 8 μl of 5% hydroxylamine was then added to the sample and incubated for 15 min to quench the reaction. After that, 11 TMT-labeled samples in the same group were pooled and lyophilized. Half of the TMT-labeled peptide mixture was fractionated using a Waters XBridge BEH130 C18 3.5 μm 2.1 × 150 mm column on an Agilent 1260 HPLC operating at 0.2 ml/min. Buffer A consisted of 10 mM ammonium formate and buffer B consisted of 10 mM ammonium formate with 90% acetonitrile; both buffers were adjusted to pH 10 with ammonium hydroxide as described previously (25). A CBS-B programed multifunction automatic fraction collecting instrument (Huxi instrument, Shanghai, China) was coupled to the HPLC to collect eluted peptides.

### Nanoflow LC-MS/MS

An online LC-MS/MS setup consisting of an EasynanoLC system and a Q-Exactive mass spectrometer (Thermo, Bremen, Germany) equipped with a nano-electrospray ion source was used for LC-MS/MS experiments. The tryptic digested peptides were loaded on a 75 μm × 200 mm fused silica column packed in-house with 3 μm ReproSil-Pur C18 beads (Dr. Maisch GmbH, Ammerbuch, Germany) and separated with a 120-min gradient at a flow rate of 300 nl/min. Solvent A contained 100% H_2_O and 0.1% formic acid; Solvent B contained 100% acetonitrile and 0.1% formic acid. The gradient was 3%−5% B, 2 min; 5%−28% B, 93 min; 28%−40% B, 16 min; 40%–90% B, 2 min; 90% B, 7 min. The mass spectrometry instrument was operated with the temperature of the heated capillary set at 320°C and the source voltage set at 2.0 kV, MS1 full scan resolution, 70,000 at m/z 200; automatic gain control target, 3 × 10^6^; maximum injection time, 50 ms. MS2 scan resolution 70,000 at m/z 200; automatic gain control target, 2 × 10^5^; maximum injection time, 120 ms. The precursor ions were fragmented by higher energy collisional dissociation (HCD) with a normalized collision energy of 33%.

### Proteomics Analysis

Raw MS data were searched using MaxQuant (version 1.5.1.0) software (26) against a human database downloaded from UniProt (UP000005640; containing 74470 entries). The search criteria were set as the following: Trypsin/P was selected as the digestive enzyme with two potential missed cleavages. Carbamidomethyl (C) was set as a fixed modification, while acetyl (protein N-term) and oxidation (M) were set as variable modifications. The false discovery rates (FDR) of peptides and proteins were both set at 1%.

### Enzyme-Linked Immunosorbent Assay

Leukotriene A_4_ hydrolase (LTA_4_H) (abx572445, Abbexa Ltd, Cambridge, UK), Mucin 5 subtype B (MUC5B) (CSB-E11201h; CUSABIO, Hubei, China), lipopolysaccharide binding protein (LBP) (DY870-05, R&D Systems Inc) and C4b-binding protein (C4BPB) (CSB-EL003954HU; CUSABIO, Hubei, China) from serum samples were measured using ELISA according to the manufacturer’s protocols. Absorbance was measured on a plate reader at 450 nm. Serum concentration of the protein of interest was quantified by extrapolation against the standard curve.

### Statistical Analysis

We used t-test and Wilcoxon signed rank test to determine significant differences of values between groups. The stepwise regression analysis was performed to identify which rhinitis-relevant parameters explained the most variability in AIT results. We applied the receiver-operator characteristic (ROC) curve and area under the curve (AUC) analyses to determine the accuracy of using LTA_4_H as a potential biomarker in a given specimen. The ROC curve is a fundamental tool for diagnostic test evaluation. AUC is defined as the area enclosed by the coordinate axis under the ROC curve. The closer the AUC value is to 1.0, the higher the authenticity of the detection method is. All P values shown were two sided, and a P value of <0.05 was considered statistically significant.

## Results

### Serum Immunoglobulin Level

**Figure 2:**
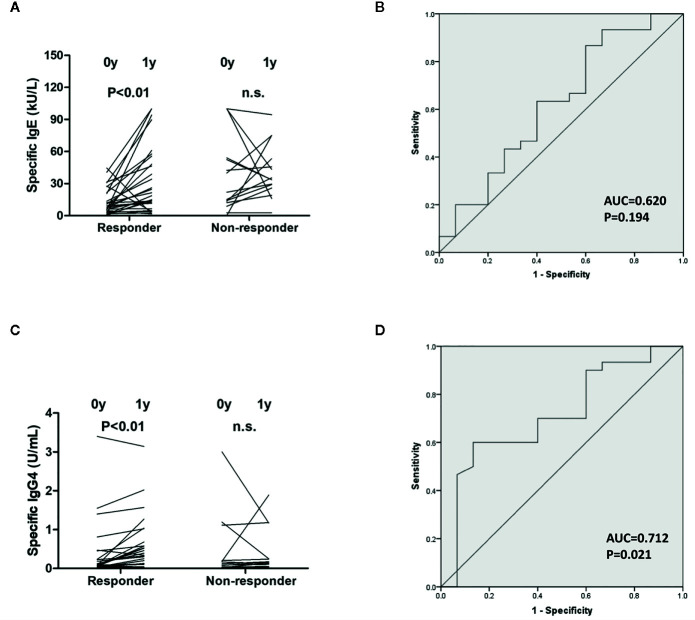
Measurements of serum immunoglobulin level. *Artemisia*-sIgE **(A)** and *Artemisia*-sIgG4 **(C)** in the sera of responders and non-responders before and after 1-year allergen immunotherapy (AIT) were measured as described in the Methods section. Receiver-operator characteristic (ROC) curve of *Artemisia*-sIgE **(B)** and *Artemisia*-sIgG4 **(D)** in the sera of responders and non-responders. n.s., not significant.

Serum immunoglobulin levels were detected in both groups before and after 1-year therapy. *Artemisia*-sIgE and *Artemisia*-sIgG4 were elevated only in the responders (P<0.01) (**Figure 2**). Furthermore, we applied the ROC curve to determine the accuracy of using *Artemisia*-sIgE and *Artemisia*-sIgG4 as potential biomarkers in our specimen. The assessment of *Artemisia*-sIgE generated an AUC value of 0.620 (95% confidence interval: 0.438 to 0.802; P=0.194) in distinguishing responders from the non-responders (**Figure 2B**). The assessment of *Artemisia*-sIgG4 generated an AUC value of 0.712 (95% confidence interval: 0.550 to 0.875; P=0.021) in distinguishing responders from the non-responders (**Figure 2D**).

### Stepwise Regression Analysis

A stepwise regression analysis, with AIT results as the dependent variable and changes of those allergic rhinitis-relevant parameters listed in **Table 1** as the independent variables, was performed to identify which rhinitis-relevant parameters explained the most variability in AIT results. As shown in **Table 2**, the final step of the regression analyses retained two of the eight predictor variables: sneeze and nasal congestion. Taken together, these two variables explained approximately 84.1% of the variability in AIT results.

**Table 2:** Regression statistics for a stepwise regression of allergic rhinitis-relevant predictors on allergen immunotherapy (AIT) results, final model.

	R^2^	Unstandardized β	Standardized β	95% CI		P
				Lower	Upper	
Model	0.841					
sneeze		0.216	0.702	0.166	0.265	<0.001
nasal congestion		0.108	0.301	0.050	0.165	0.001

### Characteristics of Serum Proteins by Quantitative LC-MS/MS

**Figure 3:**
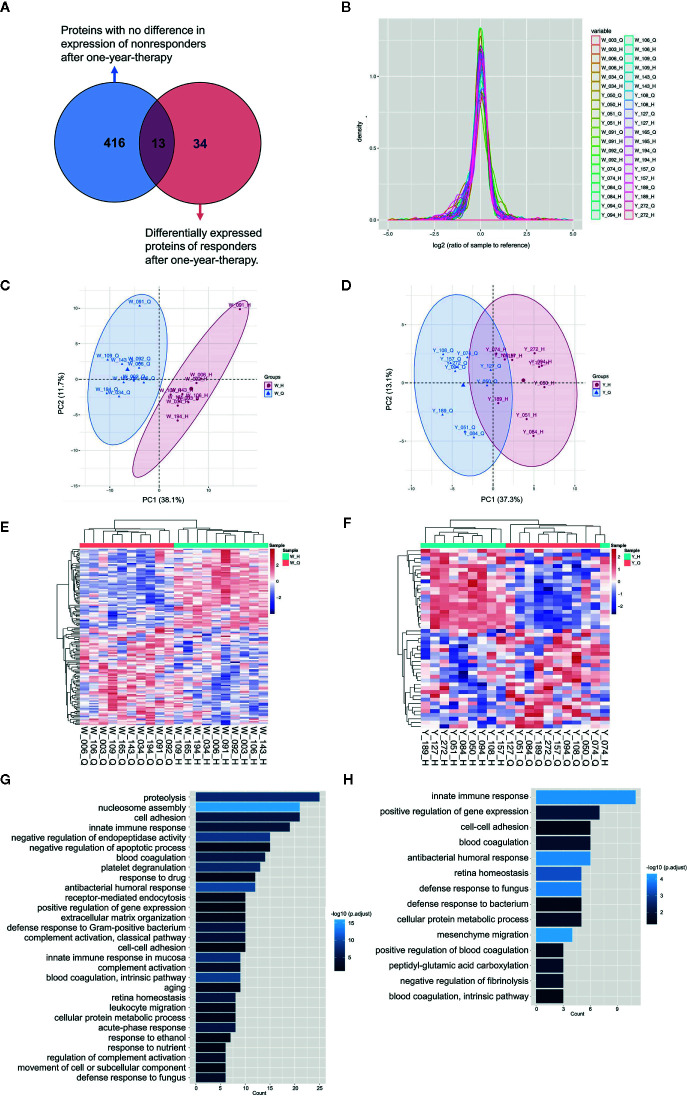
Characterization of serum proteins using quantitative MS. **(A)** Venn diagram depicts the overlap between proteins with no difference in expression of non-responders and differentially expressed proteins of responders after 1-year therapy. **(B)** The unimodal distributions of the ratios of samples to the internal reference. Principal-component analysis of serum proteins before and after 1-year allergen immunotherapy (AIT) in non-responder cohort **(C)** and responder cohort **(D)** based on tandem mass tags (TMT) global data. The heat map and Gene Ontology biological processes enrichment of the significantly differentially expressed proteins (P-adjusted < 0.05, t test) in AIT non-responders **(E, G)** and responders **(F, H)** after 1-year therapy. *W_Q: non-responder, before AIT; W_H: non-responder, after AIT; Y_Q: responder, before AIT; Y_H: responder, after AIT.

**Figure 4:**
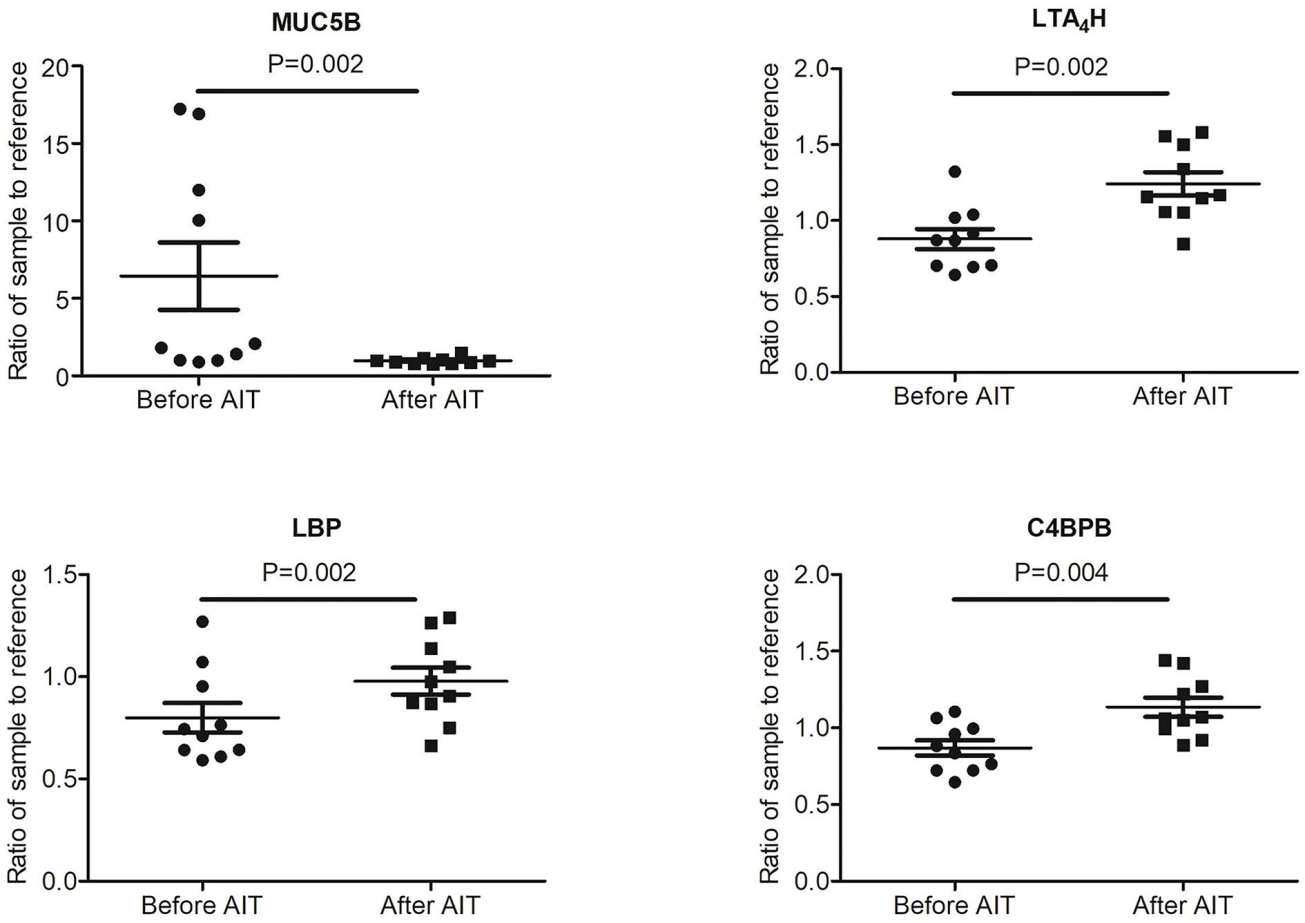
Expression level of potential biomarkers (MUC5B, LBP, C4BPB and LTA_4_H) of allergen immunotherapy (AIT) efficacy identified by MS-based proteomics in AIT responder cohort.

To identify potential biomarkers, proteomics of 10 AIT responders and 10 non-responders before and after 1-year AIT were compared. The unimodal distributions of the ratios of sample to mixture suggested no obvious degradation in serum samples (**Figure 3B**). Principal-component analysis clearly separated the serum samples before and after 1-year treatment in non-responder cohort and responder cohort based on TMT global data, and no batch effects were observed (**Figures 3C, D**). Among 573 proteins evaluated, 47 proteins were differentially expressed before and after 1-year AIT in responders and 143 proteins were identified expressed differentially in non-responders (**Figures 3E, F** and **Supplementary Table S1**) by differential analysis with P-adjust value < 0.05. The biological processes associated with those proteins were shown in **Figures 2G, H**. 13 proteins were found to have statistical difference in expression level after 1-year therapy in AIT responder cohort (P values less than 0.01) (**Table 3** and **Figure 3A**), while in non-responders their level remained unchanged. Based on their association with allergy and the protein fold change (more than 1.1 or less than 0.9) between ATI responders and non-responders, four potential genes (MUC5B, LBP, C4BPB, LTA_4_H) were identified as potential biomarkers that indicate an effective AIT. Of the four proteins, MUC5B displayed a low level of expression, while the other three (LBP, C4BPB, LTA_4_H) showed high serum levels in the AIT responders after 1-year treatment as compared with the levels before the treatment (**Figure 4**).

**Table 3:** Serum proteins with significant change of expression levels after 1-year treatment.

Protein ID	Name	Gene name	Molecular weight [kDa]		Sequence length (bp)	Uniprot ID	P	P adjust	Median of ratio After/Before AIT
Q9HC84	Mucin-5B	MUC5B	596.33	5,762		Q9HC84	0.002	0.035	0.509
B1AKG0;Q03591	Complement factor H-related protein 1	CFHR1	30.858	271		B1AKG0	0.004	0.048	0.848
O95445	Apolipoprotein M	APOM	21.253	188		O95445	0.002	0.035	0.868
P19652	Alpha-1-acid glycoprotein 2	ORM2	23.602	201		P19652	0.002	0.035	0.890
J3KPA1;P54108	Cysteine-rich secretory protein 3	CRISP3	30.975	276		J3KPA1	0.002	0.035	0.903
M0R0Q9	C3	C3	11.155	101		M0R0Q9	0.004	0.048	0.905
O75882	Attractin	ATRN	158.54	1,429		O75882	0.002	0.035	0.916
P00747	Plasminogen; Plasmin heavy chain A; Activation peptide; Angiostatin; Plasmin heavy chain A, short form; Plasmin light chain B	PLG	90.568	810		P00747	0.002	0.035	0.922
P02760;S4R3Y4	Protein AMBP;Alpha-1-microglobulin;Inter-alpha-trypsin inhibitor light chain; Trypstatin	AMBP	38.999	352		P02760	0.002	0.035	0.931
F5H810;Q99784;F5GZQ2;F8W870	Noelin	OLFM1	24.221	208		F5H810	0.004	0.048	0.937
P18428	Lipopolysaccharide-binding protein	LBP	53.383	481		P18428	0.002	0.035	1.188
P20851	C4b-binding protein beta chain	C4BPB	28.357	252		P20851	0.004	0.048	1.316
P09960	Leukotriene A-4 hydrolase	LTA_4_H	69.284	611		P09960	0.002	0.035	1.436

### Validation of LC-MS/MS Data by ELISA

**Figure 5:**
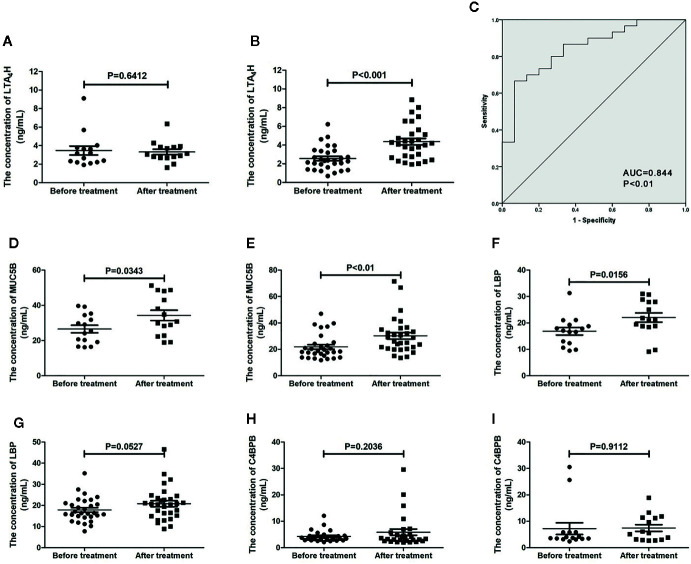
Validation of proteomic prognostic biomarkers using enzyme-linked immunosorbent assay (ELISA). The serum level of LTA_4_H in allergen immunotherapy (AIT) non-responders **(A)** and AIT responders **(B)**. **(C)** Receiver-operator characteristic (ROC) curve of LTA_4_H measured using ELISA. The serum level of MUC5B in AIT non-responders **(D)** and responders **(E)**. The serum level of lipopolysaccharide binding protein (LBP) in AIT non-responders **(F)** and responders **(G)**. The serum level of C4BPB in AIT non-responders **(H)** and responders **(I)**.

To evaluate the diagnostic value of the candidate four proteins, we measured their expression levels in 30 AIT responders and 15 AIT non-responders (**Table 1**), which were also included in the quantitative MS. The clinical responses of AIT responders and non-responders were not statistically different before AIT, enabling to control many potential confounders. Among the four candidate proteins, only LTA_4_H showed were consistent results with the proteomics data (**Figure 5**). The LTA_4_H level in the responders increased significantly (mean 1.802 ng/ml, P<0.001) after 1-year therapy, while that of non-responders remained unchanged (**Figures 5A, B**). Furthermore, we applied the ROC curve to determine the accuracy of using LTA_4_H as a potential biomarker in our specimen. The assessment of LTA_4_H generated an AUC value of 0.844 (95% confidence interval: 0.727 to 0.962; P< 0.05) in distinguishing responders from the non-responders (**Figure 5C**). The AUC value of LTA_4_H was larger than those of *Artemisia*-sIgE and *Artemisia*-sIgG4, indicating that LTA_4_H might be superior as biomarker to *Artemisia*-sIgE and *Artemisia*-sIgG4 in predicting response after 1 year of AIT. These findings imply that the serum LTA_4_H might be potentially useful in estimating the efficiency of AIT.

## Discussion

AIT is a well-established treatment scheme for IgE-mediated respiratory allergies, inducing a long-lasting reduction of symptoms in association with a down-regulation of allergen-specific TH2 responses (27–29). However, less than one third of patients are offered this therapy and up to 30% of patients do not respond to it (30, 31). Patient selection and compliance might be improved if predictive biological markers were available to identify those individuals more likely to benefit from AIT.

To address this issue, a total of 45 patients with *Artemisia* pollen allergic rhinitis were included in this study. Clinical responses and immunoglobulin levels were evaluated before and after AIT. A success AIT significantly increased *Artemisia*-sIgE and the *Artemisia*-sIgG4, which was in line with previous reports (13, 32). Regression analysis of allergic rhinitis-relevant parameters collected in this study provided a robust model that included two most significant variables (sneeze and nasal congestion) that explain 84.1% of the variability in AIT results. Allergic rhinitis-relevant parameters were based on subjective feelings of patients. Single subjective index is difficult to standardize and is easily affected by many factors. Thus, it should be used combined with objective biomarkers to predict the efficacy of AIT. This is also the reason why we want to look for serum objective biomarkers of AIT efficacy. Nasal secretion is also a good sample type to mirror the pathological status of allergic rhinitis. However, nasal secretion is less stable and easy to be polluted by the external environment, which may affect the results. The clinical collection procedure of nasal secretions is more complicated than that of blood, which may decrease the practical utility of biomarkers. Serum represents valuable resource for proteome analysis and biomarker discovery due to its availability and stability. Thus, we chose blood instead of nasal secretions as study samples.

Our study used a LC-MS/MS-based proteomics approach followed by ELISA validation to identify and confirm proteins associated with AIT results. First, we performed a comparative proteomic analysis on sera obtained from 10 AIT responders and 10 non-responders before and after 1-year treatment to select candidate biomarkers. Then we used sera from 30 AIT responders and 15 AIT non-responders to validate their diagnostic usefulness. Finally, we found that LTA_4_H is up-regulated only in AIT-responders. The ROC assessment of LTA_4_H generated an AUC value of 0.844 (95% confidence interval: 0.727 to 0.962; P<0.05) in distinguishing responders from the non-responders. The AUC value of LTA_4_H was larger than those of *Artemisia*-sIgE and *Artemisia*-sIgG4, demonstrating that LTA_4_H may have considerable accuracy in predicting AIT results. Taken together, these findings suggest the high potential value of LTA_4_H as a marker of effective AIT after the treatment of the first year. On other hand, the ELISA results of LBP, C4BPB and MUC5B are inconsistent with that of MS-based proteomics. However, they may be worthy further study for their potential as predictive markers. A previous study showed that the existence of isoforms might reduce the reliability of protein detection by antibody probes (33), suggesting there might be room to improve the quantification of these proteins with better ELISA, which may subsequently improve the consistence with MS-based proteomics.

LTA_4_H is responsible for the synthesis of Leukotrienes (LTs). LTs have been identified as central mediators in asthma and allergy (34). But we did not find significantly changes in their serum level before and after 1-year AIT through MS analysis. We speculated that their serum levels might not be influenced by AIT. Moreover, no studies have shown that they could be reliably used as a predictor of AIT efficacy. Thus, we did not consider these mediators in this study. LTA_4_H plays important roles in the recruitment of neutrophils, with disparate functions in generating one neutrophil chemoattractant (LTB_4_), while degrading another (Pro-Gly-Pro, PGP) (35, 36). Neutrophils are defined as important cells in the development of allergic sensitization and inflammation (37). They are one of the first innate immune cells recruited into the lungs during specific asthma-related events such as allergenic challenges (38). Uncontrolled allergic rhinitis and asthma are associated with elevated numbers of neutrophils (39, 40). Activated neutrophils can lead to allergic inflammation in allergic rhinitis by priming T cells and attracting eosinophils (40). Clinical data showed increased levels of neutrophils in nasal biopsies, nasal lavage fluid (NAL) and peripheral blood from patients with allergic rhinitis during the pollen season compared to healthy controls (40–42). The aminopeptidase activity of LTA_4_H with PGP as substrate is augmented in the presence of albumin (43) and chloride ions (44). Due to the disparate concentrations of chloride ions and albumin intracellularly and extracellularly, the epoxide hydrolase capacity of LTA_4_H occurs intracellularly and the aminopeptidase activity operates extracellularly. Thus, we thought serum LTA_4_H mainly operated the aminopeptidase capacity, which degraded PGP, and then restricting recruitment of neutrophils and facilitating the resolution of inflammation. The elevation of serum LTA_4_H in responders can better inhibit the progress of neutrophil driven inflammation by degrading PGP, which may be the reason for the good efficacy of AIT (45).

AIT candidate biomarkers proposed so far could be grouped into six domains: (i) antibodies, including IgE and IgG4, (ii) serum inhibitory activity for IgE, including IgE-FAB and IgE-BF, (iii) basophil activation, including CD63 and CD203c, (iv) cytokines and chemokines; (v) cellular markers and (vi) *in vivo* biomarkers (13, 46). Due to the differences in treatment course, desensitization vaccine type and allergic disease type between our studies and previous studies, we did not find the above biomarkers in our results. Previous studies often proposed some targeted candidate markers at first, and then determined the predictive value of these candidate biomarkers by single detective methods, including immunoCAP, ELISA, real-time PCR or flow cytometry (13, 46–49). In contrast, our study used a LC-MS/MS-based proteomics approach to screen candidate biomarkers in whole serum without presetting any target. Then, ELISA was performed to validate the results of proteomics, ensuring the accuracy.

Our study had a number of notable strengths. First, we had a complete set of allergic rhinitis control symptoms scoring system. All patients were evaluated on a uniform standard, enabling controls of many potential confounders. Second, our incorporation of an ELISA validation based on the initial proteomics approach allowed for confirmation of initially identified proteins and verified the robustness of our findings. This repeated analysis study design provided protection against the detection of false positive markers. Third, we found that in patients undergoing AIT, the increase of LTA_4_H level was associated with a more effective AIT. LTA_4_H level in serum may identify patients at-risk for AIT failure, enabling proactive and targeted interventions, and serum LTA_4_H level might provide a means of monitoring course of AIT. Last, these findings have the potential to lead to new studies to design and target intervention strategies that modulate the LTA_4_H level to achieve an effective AIT.

The limitations of the study were as follows: (i) it was a single-center study with relatively small sample size; (ii) the biomarker was estimated after 1-year treatment and therefore might be not able to predict the outcomes of AIT at the very beginning; (iii) due to the high drop-off rate of AIT, this study was not include placebo control and subjects were not blinded; (iv) this study could not identify late responders and sustained unresponsiveness following discontinuation of AIT; (v) it was also possible that some proteins might have degraded before analysis and would have reduced our ability to detect them in this analysis. We will continue to track the follow-up treatment of all patients.

In conclusion: using proteomics together with ELISA, we have identified serum LTA_4_H level as a diagnostic candidate biomarker for predicting AIT results. Further efforts are required to elucidate its biological functions in AIT and to confirm its diagnostic values in larger cohorts in clinic setting. Future validation of other candidates defined by proteomics in a large cohort study may help develop additional biomarkers that can be added to LTA_4_H to have more diagnostic efficacy for the successful AIT.

## Data Availability Statement

The mass spectrometry proteomics data have been deposited to the ProteomeXchange Consortium *via* the PRIDE partner repository with the dataset identifier PXD022130.

## Ethics Statement

The studies involving human participants were reviewed and approved by the Institutional Review Board of Beijing Shijitan Hospital, Affiliated to Capital Medical University. Written informed consent to participate in this study was provided by the participants’ legal guardian/next of kin.

## Author Contributions

J-FW, X-YW, and J-SY supervised the project. T-TM, J-LS and H-YS collected the sera. R-LY, W-JY collected important background information. M-DC and CP carried out the study and analyzed the data. J-GL, Q-YW, and D-YW provided assistance for data acquisition, data analysis, and statistical analysis. M-DC and T-TM wrote the manuscript. M-DC, T-TM and J-FW revised the manuscript. All authors contributed to the article and approved the submitted version.

## Funding

This study was partially supported by Beijing Municipal Science & Technology Commission (Grant No. Z161100000516006), Beijing Municipal Administration of Hospitals Clinical Medicine Development of Special Funding Support (Grant No. ZYLX201826), Science and technology research and development project of China National Railway Group Co (Grant No. J2019Z603) and the fund of Beijing Shijitan Hospital (Grant No. 2018 q-16).

## Conflict of Interest

The authors declare that the research was conducted in the absence of any commercial or financial relationships that could be construed as a potential conflict of interest.
